# A New Model for Management of Mycetoma in the Sudan

**DOI:** 10.1371/journal.pntd.0003271

**Published:** 2014-10-30

**Authors:** Ahmed Fahal, El Sheikh Mahgoub, Ahmed Mohamed EL Hassan, Manar Elsheikh Abdel-Rahman, Yassir Alshambaty, Ahmed Hashim, Ali Hago, Eduard E. Zijlstra

**Affiliations:** 1 Mycetoma Research Centre, University of Khartoum, Khartoum, Sudan; 2 Faculty of Mathematical Sciences, University of Khartoum, Khartoum, Sudan; 3 Faculty of Medicine, Bakht el Ruda University, Sudan; 4 Rotterdam Centre for Tropical Medicine, Rotterdam, The Netherlands; University of California San Diego School of Medicine, United States of America

## Abstract

Patients with mycetoma usually present late with advanced disease, which is attributed to lack of medical and health facilities in endemic areas, poor health education and low socio-economic status. With this background, an integrated patient management model at the village level was designed to address the various problems associated with mycetoma. The model was launched in an endemic village in the Sudan, between 2010 and 2013. This model is described in a prospective, descriptive, community-based study, aimed to collect epidemiological, ecological, and clinical data and to assess knowledge, attitude and practice (KAP) in order to design effective and efficient management measures. In this study, the prevalence of mycetoma was 14.5 per 1,000 inhabitants. The patients were farmers, housewives and children of low socio-economic status, and no obvious risk group was detected. All had surgery performed in a mobile surgical unit in the village which encouraged patients to present early with small early lesion leading to a good clinical outcome. The close contact with the Acacia tree thorns, animals and animal dung, walking bare footed and practising poor hygiene may all have contributed to the development of mycetoma in the village. Knowledge of mycetoma was poor in 96.3% of the study population, 70% had appropriate attitudes and beliefs towards interaction with mycetoma patients and treatment methods, and 49% used satisfactory or good practices in the management of mycetoma. Knowledge and practices on mycetoma were found to be significantly associated with age. Based on the KAP and epidemiological data, several health education sessions were conducted in the village for different target groups. The integrated management approach adopted in this study is unique and appeared successful and seems suitable as an immediate intervention. While for the longer term, establishment of local health facilities with trained health staff remains a priority.

## Author Summary

The Mycetoma Research Centre (MRC) in Sudan adopted an integrated village management model in an attempt to encourage patients to present early for treatment. The model consisted of a house to house survey to detect mycetoma suspected patients and to refer them for further management, KAP, epidemiological and ecological studies. In this study, 33 new patients were detected, and no definite risk factor was detected. However, contact with thorns, animals and animal dung may have contributed to the development of mycetoma. All patients had medical treatment and wide local surgical excisions in a mobile surgical unit at the village, except one with massive lesion who was referred to the MRC for further management. As the study population's knowledge, attitude and practice to mycetoma were poor, several health education sessions were conducted. This integrated management approach proved to be practical and successful. The various problems associated with the late presentation of patients and poor treatment outcome were addressed simultaneously at village level. That had encouraged patients to present early with small lesions with good outcome. However, for the longer term management of mycetoma patients, establishment of local medical and health facilities with qualified health staff remains essential and urgent.

## Introduction

Mycetoma is a neglected tropical medical and health problem. It is a chronic, specific, granulomatous, progressive and disfiguring inflammatory disease caused by true fungi or by certain bacteria and hence it is usually classified into eumycetoma and actinomycetoma respectively [1], [2]. *Madurella mycetomatis* is the commonest causative agent for eumycetoma, while *Streptomyces somaliensis* and *Nocardia brasiliensis* are the common causative organisms for actinomycetoma [3], [4]. Mycetoma is reported worldwide but highly endemic in what is known as the “mycetoma belt” and the Sudan seems to be the mycetoma homeland [1], [2]. The true incidence and prevalence of mycetoma world-wide is not precisely known [5], [6], [7], [8]. It is interesting to note that most of the reported mycetoma data are from hospital-based studies and from patients with advanced disease while there are few field-based observations [9], [10], [11].

The triad of a painless subcutaneous mass, sinus formation and purulent or sero-purulent discharge that contains grains is pathognomonic of mycetoma [12], [13]. It may spread to involve the skin and the deep structures, resulting in destruction, deformity and loss of function; occasionally it can be fatal. The foot and hand are the most frequently affected sites accounting for 82% of cases. In endemic areas other parts of the body may be involved such as the knee, arm, leg, head and neck, thigh and perineum [14]. No age is exempted in mycetoma; however, it occurs more frequently in young male patients in the age range 20–40 years [1], [5]. Most of the affected patients are farmers and workers of low socio-economic status [3], [4], and almost 30% of reported patients are children [15].

Currently, diagnostic tools include various imaging techniques and methods to demonstrate the organism e.g. by molecular techniques such as PCR or by culture; there is no reliable serodiagnosis test. A histo-pathological specimen is useful but definite identification of the pathogen is not always possible [16], [17], [18], [19], [20]. It is important to note most of these techniques are not available in majority of mycetoma endemic regions.

Patients tend to present late with massive lesions. This is attributed to the nature of mycetoma which is usually painless and slowly progressive, the lack of health facilities in endemic areas, the low socio-economic status of the affected patients and their poor health education [1]–[5]. For this reason, the current treatment of mycetoma is suboptimal, characterised by low cure rates and frequent recurrence often leading to amputation [21]. However, clinical experience shows that early and small mycetoma lesions are associated with good outcome and prognosis. Presently, there are no prevention and control measurements for mycetoma [22], [23].

The Mycetoma Research Centre (MRC) was established in 1991 under the auspices of the University of Khartoum, based at Soba University hospital to provide high quality medical care, research, education and teaching in the various aspects of mycetoma and to provide integrated community development activities in endemic areas. Since its establishment, more than 6800 patients were seen and treated at the centre (www.mycetoma.edu.sd).

In an attempt aimed at improvement of case detection with early diagnosis and thus better outcome, the MRC has developed a new and innovative integrated management approach that addresses various problems associated with mycetoma at the village level. In this communication, we report on this integrated comprehensive management experience in a village in the endemic area for mycetoma in Sudan.

## Materials and Methods

This prospective, descriptive, community-based study was conducted at the Al Andalous village, at the White Nile State, Sudan. The village was selected as in recent years, MRC records showed high incidence of mycetoma from that village. The study was conducted between June 2010 and June 2013 and included all the village inhabitants. Epidemiological and ecological aspects of the disease, clinical presentation of patients with mycetoma and the villagers' knowledge, attitude and practice (KAP) towards mycetoma were investigated.

### Data collection

Data were collected on demographic and ecological characteristics as well as clinical aspects of patients with mycetoma; the KAP data were collected using a closed-ended questionnaire. A pilot study was conducted in the two weeks preceding the survey to validate the data collection forms and questionnaire in a similar village (Al Firdoos), 30 km south of Al Andalous village. The surveyors had been trained in data collection before the study.

The study was carried out by the Mycetoma Research Centre, University of Khartoum, in collaboration with the Mycetoma Control Programme of the Ministry of Health of Sudan and the University of Bakht-El-Ruda Medical School at Ed Dueim. The capital of White Nile state situated close to the endemic area.

### Demographics and ecology

The studied village was divided into three clusters and every house in these clusters was visited by a team of three surveyors. The head of the household was interviewed and the details of household members were recorded. A household was defined “as people living under one roof preparing and eating together the same food”.

The village's ecological and geographic characteristics were recorded including the type of soil, trees, plants, houses and animals and water supply. The meteorological information was obtained from the Sudan Meteorological Authority, 2010.

### Mycetoma patients: Clinical aspects

A house to house survey was carried out and all suspected patients were referred to the village health centre to be examined for the presence of mycetoma by a team consisting of two consultant surgeons, two surgical registrars and two senior house officers in surgery. During the study period, four three-day clinics were conducted.

The patients' demographic characteristics were recorded and all suspected patients had a fine needle aspiration for cytology and grains culture. All patients underwent wide local surgical excisions under general or spinal anaesthesia in a mobile surgical unit based at the village health centre and all surgical biopsies were histo-pathologically examined. All patients were followed up during the study period by the surgical team and in between visits, by the medical officer from the health centre and a medical assistant. All diagnostic procedures and treatment was provided free of charge and any other illness detected among household members during the village survey were managed on a similar basis.

### The Knowledge, Attitude and Practice (KAP) study

Households were divided proportionately at a ratio of 1∶1, male to female; the male head of the household or senior female in the same household. The selected member from each household was interviewed by a team of three surveyors. A direct interview technique was used. The survey included 402 households.

The knowledge of mycetoma referred to the understanding of the concepts of mycetoma that related to mode of transmission, risk groups, symptoms, diagnosis, treatment and prevention. This section consisted of 22 statements that were scored 1 or 0 for a correct or incorrect answer respectively. Scores were summed for each respondent and levels of knowledge were categorized as poor [1–5], mild [6–10], satisfactory [11–15] and good [>15].

The attitudes to mycetoma referred to the degree of positive or negative agreements with statements concerning attitudes and beliefs in the interaction with mycetoma patients as well as appropriate treatment methods. There were 4 statements that were scored 1 or 0 for positive or negative attitude, respectively. The levels of attitude scores were summed for each respondent and grouped into five categories as totally negative [0], mild [1], satisfactory [2], good [3], totally positive [4].

The section on practice related to mycetoma consisted of 7 statements that refer to food consumption, shoe wearing as well as other habits. The statements were scored 1 or 0 for good or poor practice, respectively. The levels of practice scores were summed for each respondent and grouped into four categories as poor [0–1], mild [2–3], satisfactory [4–5] and good [6–7].

### Health education

During the study period and following analysis of the KAP data, several health education sessions on mycetoma were conducted. The sessions were delivered by well-trained medical students and volunteers. In addition, health education materials were prepared and distributed during the sessions.

### Patients follow up

All patients were followed up during the study period for evidence of recurrence by the surgical team and the health centre staff.

### Statistical analysis

Data were managed using the epidata software, incorporating appropriate skips and range checks. Stata statistical software version 12 was used for analysis.

### Ethics statement

Written informed consents were obtained from the local health authority, village leaders and the individual study participants and an ethical clearance was obtained from Soba University Hospital Ethical Committee.

## Results

### Demographics

The village is in White Nile State, Sudan; a known mycetoma endemic area it is 250 kilometres south of the capital Khartoum with a population of 2835 inhabitants divided over 405 households. All the residents are from one ethnic group; the Hassania tribe. The villagers were mainly agriculturalists and pastoralists. They have farms just outside the village with irrigation from the White Nile and the main crops included sorghum, wheat, cotton and vegetables. In addition, they rear cattle, goats and sheep. Other animals in the village included chickens, donkeys and dogs.

**Figure 1:**
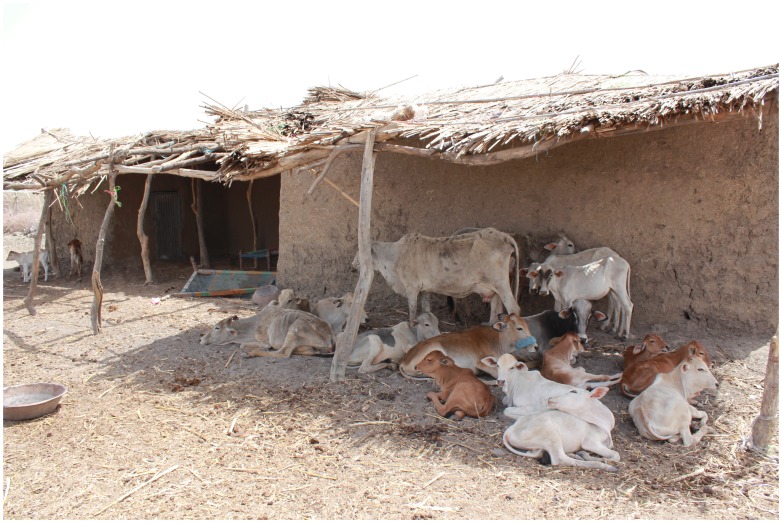
Showing a typical house made of mud with roofs made of tree branches with various animals living on the compound.

The village has two parts; an older part with poor hygiene, where houses are overcrowded and where often many animals are kept inside the compound or in a fenced area made of Acacia thorny branches that surround the houses. The animals were sheep, cattle, goats, chicken, donkeys, camels and dogs (Figure 1). As a consequence the ground is covered with a layer of animal dung. In the newer part of the village, the houses were separated from each other, less crowded and have better hygiene; in only some of the houses animals were kept in the same compound. The village has small health centre staffed by one doctor, one laboratory technician, one medical assistant and four nurses.

### Ecology

There were different types of houses; while some were made of bricks and mud, others were from tree branches. In most houses, the ground was covered with straw, thorns and animal dung.

**Figure 2:**
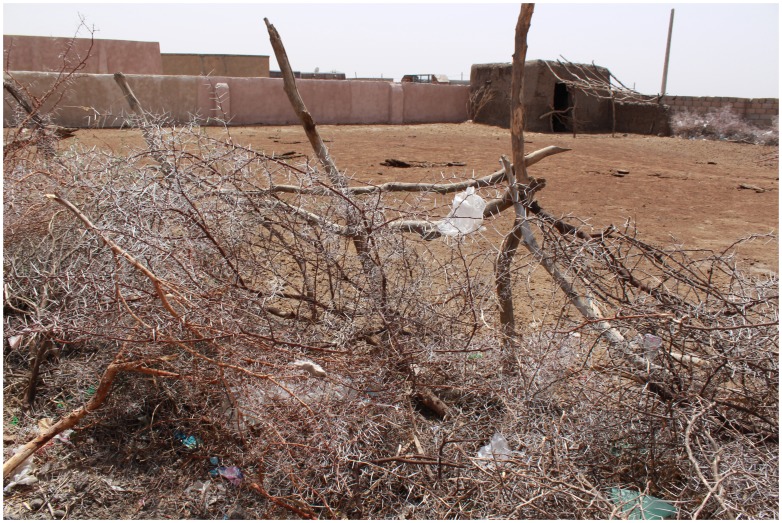
Showing an enclosure demarcated with Acacia tree branches to keep animals or hay.

Different types of trees were found in the village and its immediate surroundings and that included *Acacia senegal*, *Acacia seyal*, *Acacia nilotica as well as Zizyphus spp.* and *Balanites aegyptiaca*. People used branches of Acacia to demarcate areas for storage of hay or to keep animals (Figure 2).

The village has a loamy soil similar to other mycetoma endemic Sudanese states such as Gezira and Sennar States. Water is provided by a central pump and distributed by donkey cart.

The daily temperatures in the dry season range from 19–43°C with relative humidity of 21–38%. There is a short but heavy rainy season between June and September with the monthly rainfall reaching 136 mm; in this rainy season the daily temperature ranges from 34–41°C and humidity 60–70% (Sudan Meteorological Authority, 2010, unpublished data).

### Prevalence of mycetoma

In the studied village, malaria, schistosomiasis and mycetoma were the three most common health problems with mycetoma ranking third. The prevalence of mycetoma was 14.5/1000 inhabitants. Most patients were from the older part of the village with the prevalence of 8.3/1000 which is higher than recorded in the newer part of the village (6.2/1000), but the difference did not reach statistical significance (p = 0.07).

### Mycetoma patients: Clinical aspects

The study included 33 patients with confirmed mycetoma; 16 (49%) were males. The age ranged between 11 and 70 years with a median of 23 years and mean 30 years ±0.25 SE). The majority [22, (67%)] were <30 years, (Table 1). In this study, school children 11 (33%) were affected most, followed by farmers 8 (24%). One patient (3%) was unemployed because of the prolonged illness and disability. Housewives constituted 21% of the affected patient population. Most patients were born and lived in the village; those who lived outside the village were living in nearby villages in the same State. (Table 1)

**Table 1:** Demographic characteristics of 33 mycetoma patients diagnosed at Al Andalous village, White Nile State, Sudan.

The Demographic Characteristics	No.	Percent
**Sex**		
Male	16	48.5
Female	17	51.5
**Age**		
<20	11	33.3
20<30	11	33.3
30+	11	33.3
**Occupation**		
Student	11	33.3
Farmer	8	24.2
Worker	4	12.1
House Wife	7	21.2
Jobless	1	3.3
Others	2	6.6
**Duration in Years**		
<1	11	33.3
>1–5	14	42.4
>5–10	2	6.1
>10–20	3	9.1
>20–30	2	6.1
30+	1	3.0
**Pain**		
Yes	19	57.6
No	13	39.4
Missing	1	3.0
**Trauma**		
Yes	16	48.5
No	6	18.2
Not Sure	11	33.3
**Concomitant medical problems**		
Yes	1	3.3
No	32	96.7
**Family history**		
Yes	17	51.5
No	16	48.5
**History of previous surgical excision**		
Yes	17	51.5
No	16	48.5
**Lesion site**		
Foot	28	85
Hand	4	12
Gluteal	1	3
**Lesion diameter**		
<5 cm	12	36.4
5–10 cm	9	27.3
>10 cm	12	36.4
**Lesion sinuses**		
None	15	45.5
Active	6	18.2
Healed	12	36.4

The disease duration ranged between 0.17 and 50 years with a median of 2 years and mean 7 years ±0.3 SE). While the disease onset, course and progress were typical, it was remarkable that 12 patients had small lesions (<5 cm) of more than 5 years duration.

Sixteen patients (48%) had previous surgical excisions and recurrence. Seventeen patients (52%) had family history of mycetoma and only one patient had diabetes as a concomitant medical problem (Table 1).

**Figure 3:**
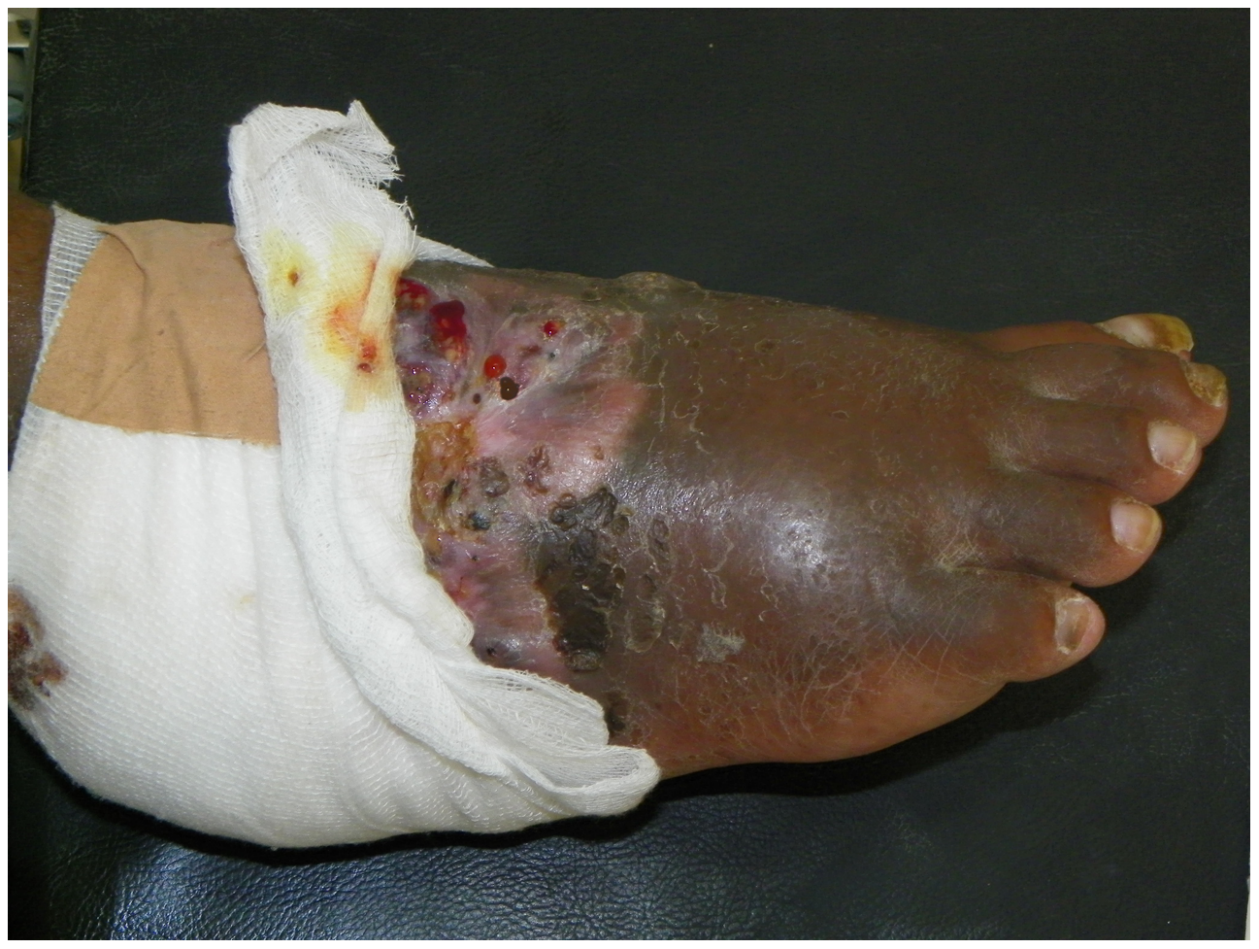
Showing an early lesion, with no sinuses in one of the patients in the village.

**Figure 4:**
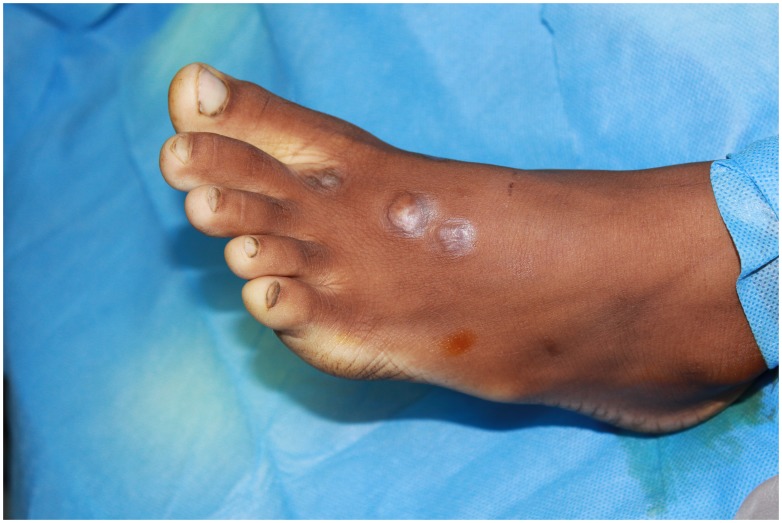
Showing classical mycetoma on the foot with sinuses discharging black grains in one of the patients.

**Figure 5:**
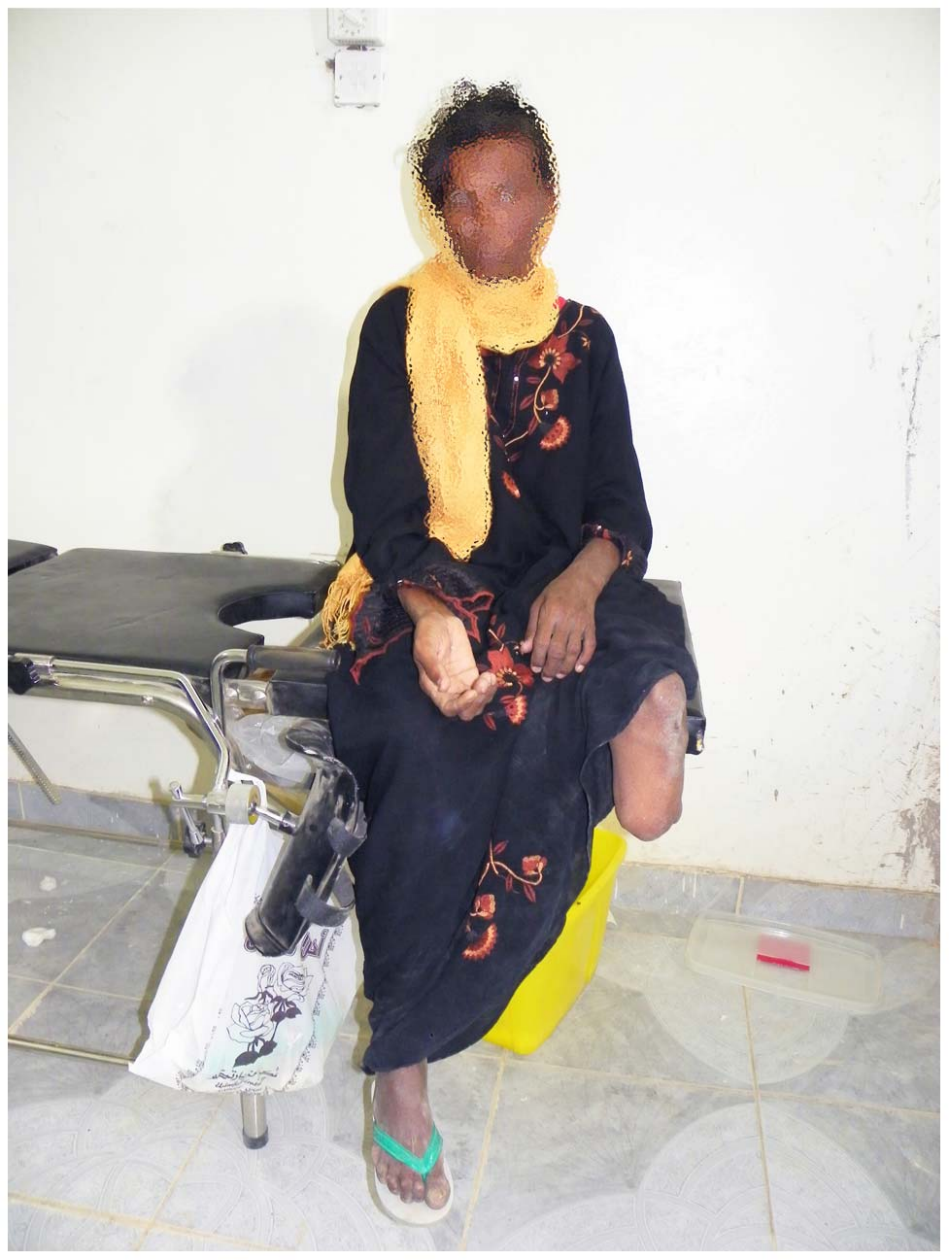
Showing a woman with recurrent the right hand mycetoma and a left below knee amputation due to mycetoma.

The foot was most frequently affected seen in 28 (85%), followed by the hand in 4 (12%) and gluteal region in one (3%). The lesions ranged between small (<2 cm, n = 12), moderate (5–10 cm, n = 9) and massive (>10 cm, n = 12). Six of the patients had lesions with active sinuses, 12 patients had healed sinuses while 15 patients did not have sinuses. (Figures 3, 4, 5)

In the village, there were another eight patients with past history of mycetoma and they had been cured by wide surgical excisions (n = 6) and limb amputation (n = 2).

All patients underwent wide local surgical excisions except one patient who had a massive lesion, who was referred to MRC for further management. In three patients, thorns were identified during surgical excisions. All patients had uneventful post-operative recovery.

The histopathological examination suggested the diagnosis of eumycetoma due to *M. mycetomatis* in all of patients. All patients were followed up regularly with a mean duration of 1±0.5 year with no evidence of recurrence.

### The KAP study

#### Demographic characteristics of the study population

161 (40%) of the 403 household respondents were males and 241 (60%) were females with 239 (59%) in the age group between 20–40 years and 110 (27.4%) >40 years of age. The education level assessment showed that 103 (26%) were illiterate or obtained Khalwa (basic religious) education, 195 (49%) had received basic education, while 97 (24.1%) had completed secondary school or higher education.

The study further showed that, 197 (49%) of the respondents were housewives and 94 (23%) were farmers/herders. Most (n = 350 [86.9%]) had an income <500 Sudanese pounds (about 100 USD) per month.

#### Mycetoma knowledge

Overall, 254 (63%) of the respondents demonstrated mild knowledge of mycetoma, 126 (31%) had poor knowledge while only 15 (4%) had satisfactory knowledge and none had good knowledge (Table 2).

**Table 2:** Knowledge, Attitude and Practice towards mycetoma among Al Andalous village's population, White Nile State, Sudan.

Knowledge of Mycetoma	Grade	Number (N = 403)	Percent
	Poor [1–5]	126	31.3%
	Mild [6–10]	254	63.0%
	Satisfactory [11–15]	15	3.7%
	Missing	08	2.0%
**Attitude to Mycetoma**			
	Totally negative [0]	14	3.5%
	Mild [1]	16	4.0%
	Satisfactory [2]	92	22.8%
	Good [3]	130	32.3%
	Totally positive [4]	151	37.5%
**Practice related to Mycetoma**			
	Poor [0–1]	27	6.7%
	Mild [2–3]	180	44.7%
	Satisfactory [4–5]	132	32.8%
	Good [6–7]	64	15.9%

#### Sources of information

The study showed that 391 participants (97.3%) heard of mycetoma. The source of information were relatives and friends (58.2%), mycetoma patients (32.2%), doctors (5%), villagers (3.5%) television (2.2%), radio (2%), magazines and journals (0.5%) or health assistants (0.2%). It was noted that participants had heard about mycetoma from more than one source (Table 3).

**Table 3:** Questions on knowledge on mycetoma used in the survey on Knowledge, Attitude and Practice (KAP) among the population of Al Andalous village, White Nile State, Sudan.

Statement	Yes No.(%)	No No.(%)	Not sure No.(%)
Have you heard of Mycetoma	391(97%)	9(2.2%)	3(0.7%)
Heard about the disease from: TV	9(2.2%)	371(92.1%)	23(5.7%)
Heard about the disease from: Doctor	20(5%)	360(89.3%)	23(5.7%)
Heard about the disease from: Heath assistant	1(0.2%)	379(94%)	23(5.7%)
Heard about the disease from: Nurse	00	380(94.3%)	23(5.7%)
Heard about the disease from: Mycetoma patients	130(32.3%)	251(62.3%)	22(5.5%)
Heard about the disease from: Relatives and friends	234(58.1%)	158(39.2%)	11(2.7%)
Heard about the disease from: Newspapers	2(0.%5)	378(93.8%)	23(5.7%)
Heard about the disease from: Others	24(6%)	356(88.3%)	23(5.7%)
Heard about the disease from: village people	14(3.5%)	389(96.5%)	00
People get Mycetoma from: Food	1(0.2%)	380(94.3%)	22(5.5%)
People get Mycetoma from: Drink	00	380(94.3%)	23(5.7%)
People get Mycetoma from: Skin wounds	9(2.2%)	371(92.1%)	23(5.7%)
People get Mycetoma from: Air	1(0.2%)	379(94%)	23(5.7%)
People get Mycetoma from: Insects	9(2.2%)	371(92.1%)	23(5.7%)
People get Mycetoma from: Thorns	205(50.9%)	181(44.9%)	17(4.2%)
People get Mycetoma from: Interaction with patients	19(4.7%)	362(89.8%)	22(5.5%)
People get Mycetoma from: Inheritance	2(0.5%)	378(93.8%)	23(5.7%)
People get Mycetoma from: Stepping over stones	28(6.9%)	353(87.6%)	22(5.5%)
People get Mycetoma from: Stepping over animal dung	53(13.2%)	326(80.9%)	24(6%)
People get Mycetoma from: Evil	2(0.5%)	378(93.8%)	23(5.7%)
People get Mycetoma from: Bathing in ponds	3(0.7%)	376(93.3%)	24(6%)
People get Mycetoma from: Wearing patient's shoes	45(11.2%)	335(83.1%)	23(5.7%)
People get Mycetoma from: Don't know	93(23.1%)	294(73%)	16(4%)
People get Mycetoma from: Not wearing shoes	5(1.2%)	398(98.8%)	00
People get Mycetoma from: Others	22(5.5%)	358(88.8%)	23(5.7%)
Symptoms of Mycetoma: Odema of affected part	118(29.3%)	1(0.2%)	284(70.5%)
Symptoms of Mycetoma: Pain in affected part	73(18.1%)	00	330(81.9%)
Symptoms of Mycetoma: Openings in affected part	16(4%)	00	387(96%)
Symptoms of Mycetoma: Grains out affected part	212(52.6%)	00	191(47.4%)
Symptoms of Mycetoma: Fever	(7) (1.7%)	00	396(98.3%)
Symptoms of Mycetoma: General fatigue	10(2.5%)	00	393(97.5%)
Needed lab investigation: X-ray	38(9.4%)	247(61.3%)	118(29.3%)
Needed lab investigation: ultrasound	2(0.5%)	281(69.7%)	120(29.8%)
Needed lab investigation: Take a sample	79(19.%6)	204(50.6%)	120(29.8%)
Is Mycetoma contagious/infectious	307(76.2%)	60(14.9%)	36(8.9%)
How do you think you can avoid getting Mycetoma	43(10.7%)	344(85.4%)	16(4%)

#### Knowledge on mode of infection

The study population believed that the causes of mycetoma were a thorn prick (51%), stepping on animal dung (13.2%), wearing open shoes (11.2%), stepping on stones (7%) or from direct contact with mycetoma patients (4.7%). Less common causes mentioned included transmission by air (0.2%), food (0.2%), skin wounds (2.2%), insects (2.2%), bathing in ponds (0.7%) and inheritance (0.5%).

The majority of the respondents 307 (76.2%) believed that mycetoma is infectious; 147 (36.6%) thought that mycetoma is usually contracted in autumn, while 34 (8.5%) and 22 (5.5%) believed this was in summer and winter, respectively. The majority of respondents (192 [47.8%]) believed that farmers are most prone to develop mycetoma, while 116 (28.9%) and 24 (6%) thought that herders and school children were at increased risk, respectively. (Table 3)

#### Knowledge of clinical presentation

Swelling was thought to be a presenting feature of mycetoma in 118 (29.4%) of the respondents, while 73 (19.2%) thought pain is a common feature and 16 (4%) recognized that sinuses are a feature of mycetoma; 212 (52.7%) were aware that mycetoma discharges grains and 186 (87.7%) knew the colour of the grains is black. Few considered fever (1.7%) and fatigue (2.5%) as symptoms of mycetoma (Table 3).

#### Knowledge of investigation for mycetoma

Only 38 (9.5%) respondents were aware that X-ray examination is needed for the diagnosis, two (0.5%) agreed that ultrasound examination is needed, 79 (19.6%) believed that blood testing was important, 14 (3.5%) knew that culture is needed for the diagnosis while 101 (25.1%) did not know any tool for diagnosis.

#### Knowledge on prevention

Wearing shoes was assumed by 149 (37.1%) important to prevent mycetoma infection, 25 (6.2%) believed that good hygiene can prevent it, 18 (4.5%) thought clean housing can prevent mycetoma while only one (0.2%) believed immunization can prevent the disease (Table 3).

#### Attitudes related to mycetoma

Overall, 37.6% had a totally positive attitude towards mycetoma, 32.3% had good attitude, 22.9% had satisfactory attitude, 4% had mild attitude while only 3.2% had a totally negative attitude (Table 3).

368 (91.5%) of the respondents believed that medical treatment and not the traditional treatment is the best treatment option, 347 (86.3%) preferred it because the traditional one is not effective and 303 (75.4%) thought that the public hospitals are the best place to treat the patients. Only five (1.2%) respondents believed in religious treatment.

The study showed that 164 (40.8%) of the respondents thought avoiding contact with a mycetoma patient will prevent acquiring the infection. The majority (284 [70.6%]) believed that there is no need to isolate the mycetoma patients and 167 (41.5%) will accept the diagnosis of mycetoma.

#### Practices related to mycetoma

In most of the participants, practice was summed as mild in 44.8%, satisfactory in 32.8%, good in 15.9% and poor in 6.5%. (Table 2)

Most of the respondents 302 (75.5%) believed that the mycetoma patients do not need special type of food and 314 (78.1%) thought that mycetoma patients do not need to avoid any specific type of food.

Regarding the most credible persons to provide information about mycetoma, most of the respondents 332 (82.6%) reported that a nurse is the most credible one and 40 (10%) of them thought a doctor should be the person providing the information. The majority of the respondents 255 (63.4%) reported that many members of their families walk barefooted either at home, in the street or while working in the field.

The study showed no statistical significant association between knowledge, attitudes or practices of mycetoma and gender (*p* = 0.139, 0.226 and 0.760, respectively), educational level (*p* = 0.460, 0.152 and 0.372, respectively) and income (*p* = 0.332, 0.896 and 0.414, respectively). Age was significantly associated with knowledge and practice of mycetoma (*p* = 0.018 and 0.001, respectively) while this association of age was not observed with attitude. Occupation was not associated with knowledge or attitude toward mycetoma (*p* = 0.260 and 0.212, respectively) but it was significantly associated with practice (*p* = 0.002).

## Discussion

Mycetoma is a neglected tropical disease that affects poor communities; patients usually present late with advanced disease which results in several medical, health and socio-economic problems. Hence mycetoma has a negative impact on patients, families, communities and health authorities in endemic areas.

In an attempt to address these problems, the MRC adopted an integrated village management model. It investigated the factors that obstruct patients' early presentation and the various medical, health and social consequences of mycetoma and provided immediate solutions at village level. In addition, this study is the first comprehensive epidemiological and ecological survey on mycetoma in an endemic area in Sudan.

The prevalence of mycetoma reported is higher than previously published from the Sudan, Mexico, Senegal, West Africa and elsewhere [1], [6], [7], [8], [11], [12], [13], [14]. This prevalence is probably more accurate as this study was field-based and involved screening of all inhabitants. Hospital-based studies are subject to bias as to the population who are able to seek medical advice while these factors are eliminated by the model of care described. These obstacles include the nature of mycetoma which is usually painless, slowly progressive and the patients' poor health education and financial constraints. Amputation, still the sole treatment for advanced disease in many centres, is feared by patients and may be a contributing factor as well. In many mycetoma endemic areas, the medical records and statistical information are deficient and hence the lack of adequate data on mycetoma incidence and prevalence. All these factors may have contributed to the globally reported low incidence and prevalence rates of mycetoma.

In general, males are predominately affected in mycetoma with a male to female ratio of 5∶1 in most reports [1], [6], [7]; however, in this study, the gender ratio (M/F) is 1∶ 1.1 suggesting universal and equal exposure. This seems supported by the high prevalence of mycetoma in school children (33.3%).

The majority of the villagers are farmers and workers who are engaged in animal husbandry, similar to other mycetoma endemic areas in the mycetoma belt [9]–[11]. There are many cattle, goats, sheep and other domestic animals which are kept inside their homes or in enclosures in close proximity to their homes and surrounded by a fence of dry Acacia thorny bushes. The floors of these enclosures and homes are covered with dry animals dung, thorns and trash. The practice of walking barefooted in the village is quite common and as a consequence the population is frequently exposed to thorns pricks. All these factors may contribute to the development of mycetoma. Currently it is unclear what the role of animals could be; the fungus may be excreted in the dung or this dung may promote or maintain fungal growth naturally occurring in the soil. This is supported by the observation that mycetoma was more common in the older, less hygienic part of the village.

However, if this hypothesis on the causation and route of infection is true, then the natural infection is expected to be more frequent than the reported ones. Furthermore, many patients as in this study did not recall a history of local trauma at the mycetoma site. Nevertheless, this merits further investigations to ascertain the route of infection and susceptibility to mycetoma as many observations support the hypothesis that eumycetoma is primarily environmentally acquired and suggest that *M. mycetomatis* needs special conditions for growth, as direct isolation from the environment so far has not been possible [22].

It is unclear whether individuals in endemic areas can develop subclinical infection and subsequent protective immunity without developing clinical disease. This gap in our knowledge is primarily due to the absence of accurate diagnostic tests and further studies are needed to estimate the incidence of subclinical infection and to provide clues on the susceptibility to mycetoma. The role of co-infection such as schistosomiasis that is common in the village, warrants investigation [23].

The role of genetic susceptibility is unclear; half of the patients reported to have a family member with mycetoma; whether this is the result of sharing the same epidemiological risk factors or genetic or familial factors should be investigated. This is also demonstrated currently in the lack of progression of mycetoma in some patients; 63.6% of the patients had lesions of less than 10 cm in diameter and 45.5% had no sinuses, while the median disease duration was two years and as a consequence the diagnosis of mycetoma was not always clinically suspected.

The KAP study analysis showed that most of the respondents were females as most of the households' heads were farmers and herders that were engaged in field activities outside the village at the survey time. Most of the study population proved to have low education level and of low socio-economic status which clearly reflected in their knowledge, attitude and practices related to mycetoma.

In the studied village, malaria, schistosomiasis and mycetoma were the three common health problems and mycetoma ranked third. Surprisingly, despite this, the population knowledge, attitude and practice related to mycetoma was not satisfactory and this necessitates the need for structured and well planned health education programmes in the village.

Despite that, most of the respondents (96.3%) were aware of mycetoma, only few (3.7%) had satisfactory in depth knowledge of the disease. This is in line with a KAP study on leprosy reported from endemic areas in the Sudan [24], where the knowledge about the pathological cause of leprosy was lacking but the clinical manifestations were well recognized as there were a considerable numbers of patients in the community.

It is interesting to note that, the most important source of information among the study population was relatives and friends and this may explain the poor in depth knowledge encountered in this study.

The study showed that most of the respondents had positive attitudes and beliefs with regard to interaction with mycetoma patients. Due to the concept of the extended families in the Sudan, the mycetoma patients are looked after by their families and that may contribute to the positive attitude to the patients seen in this study. This is quite different from the negative attitude of various populations to other chronic inflammatory disease with deformity and disability such as leprosy and tuberculosis [24], [25], [26]. This can be explained by the fact that these diseases are contagious and the deformities are more general whereas mycetoma is not infectious and more localized.

Most respondents believed that medical treatment in public hospitals is the best option as most had tried native and traditional treatments for many medical conditions including mycetoma without much success. However, medical treatment for mycetoma proved to be expensive or not available in the endemic regions and patients need to travel to specialised centres for treatment. Patients in endemic areas need medical and health support and this model is a good option in providing care.

Practices with regard to role of food and wearing shoes clearly need improvement; and an intervention by providing shoes may be considered as the foot is most commonly affected.

The clinical management at field level proved adequate; diagnosis could be made by FNA with confirmation later by culture or biopsy result. All patients had wide surgical excisions with uneventful post-operative outcome. While mycetoma patients often present with long standing and massive deformity and disability, it is clear that, this new management model was useful in detecting small lesions amenable to treatment with good prognosis [27], [28]. On the other hand, the study detected patients with long standing and massive disease and one patient had multiple recurrent lesions and amputation.

In endemic areas, screening programmes to increase public awareness of mycetoma and to enhance early detection are mandatory. Such programmes should include self-examination, clinical examination performed by health professionals and timely referral to specialised centres. These measurements should be adopted as they will reduce the disease and treatment morbidities.

### The way forward

Currently there is no control or preventive programme in mycetoma. In order to achieve disease control, many questions need to be answered and these include identification of the organisms involved, estimation of the incidence rate of clinical and subclinical infection and ecological studies that focus on transmission risk and infection route. These were among the objectives of this long term longitudinal study and will be the subject of further surveys in the near future.

Health education is clearly needed and the use of media such as the radio, television, newspapers, journals and brochures are important tools. However, in this study, the media was not a major information source due to the lack of objective and well-planned health educational programmes addressing mycetoma issues. Furthermore, the majority of the population were illiterate and of low socio-economic background and the television, newspapers and journals are a luxury and unaffordable. However, the use of mobile phones is being explored more and more in patient management and health education and perhaps this approach may be valuable for mycetoma as many individuals in the studied village had mobile phones.

Many studies showed that mycetoma commonly affects school children [3], [4], [15], therefore there is a critical need for targeting health messages through schools and teachers in order to reach this most susceptible group. This will empower the school children with the basic knowledge and skills which will ultimately protect them from acquiring mycetoma; this was achieved in this study.

Medical and health professionals were the least information source in this study, despite the fact that primary health care represents the core of health service in this country; better training and supervision are important.

In this study, medical students from the local Bakht el Ruda University have actively participated in conducting the survey and health education programme in the village. This is to be encouraged as it helps to raise the population awareness and provides health education; it obviously constitutes an opportunity to train medical students in the various community development activities and issues.

In conclusion, the integrated management approach adopted in this study is unique and appeared successful; the various problems associated with mycetoma were addressed simultaneously: epidemiology, ecology, KAP in the community and immediate patient diagnosis and management, including follow-up. This was achieved in a joint effort by the specialist team and the local community.

We propose this management model for immediate implementation in mycetoma endemic areas in the short term, while for the longer term, strengthening of the local health services which includes training of health workers and providing improved infrastructure with adequate diagnostic and treatment facilities should be a priority. The model may be applicable to other neglected tropical diseases.

## Supporting Information

Checklist S1 **STROBE checklist.**
(DOC)

## References

[1] Mahgoub ES, Murray IG (1973) Mycetoma. London, William Heinemann, Medical Books Ltd: pp. 1–50

[2] Fahal AH (2011) Mycetoma. Review article, Khartoum Med J 4 (1) 514–523.

[3] Fahal AH (2004) Mycetoma thorn on the flesh. Review article. Trans R Soc Trop Med Hyg 98 (1) 3–11. 14702833 10.1016/s0035-9203(03)00009-9

[4] Ahmed AO, van Leeuwen W, Fahal A, van de Sande W, Verbrugh H, et al (2004) Mycetoma caused by Madurella mycetomatis: a neglected infectious burden. Lancet Infect Dis 4 (9) 566–74. 15336224 10.1016/S1473-3099(04)01131-4

[5] Fahal AH, Hassan MA (1992) Mycetoma. Br J Surg 79 (11) 1138–1141. 1467883 10.1002/bjs.1800791107

[6] Ahmed AOA, van de Sande WW, Fahal A, Bakker-Woudenberg I, Verbrugh H, et al (2007) Management of mycetoma: major challenge in tropical mycoses with limited international recognition. Curr Opin Infect Dis 20 (2) 146–51. 17496572 10.1097/QCO.0b013e32803d38fe

[7] van de Sande WW (2013) Global Burden of Human Mycetoma: A Systematic Review and Meta-analysis. PLoS Negl Trop Dis 7: e2550. 24244780 10.1371/journal.pntd.0002550 PMC3820768

[8] Fahal AH (2013) Mycetoma in Williams, Bulstrode, O'Connell, Bailey and Love's Short Practice of Surgery 26E: 26th Edition, Oxford University Press, pp 84–88.

[9] Abbott P (1956) Mycetoma in the Sudan. Trans R Soc Trop Med Hyg 50: 11–24. 13299347 10.1016/0035-9203(56)90004-9

[10] Lynch JB (1964) Mycetoma in the Sudan. Ann R Coll Surg Engl 35: 319–340. 14248320 PMC2311794

[11] Mahgoub ES (1977) Mycoses of the Sudan. Trans R Soc Trop Med Hyg 71: 184–188. 888164 10.1016/0035-9203(77)90003-7

[12] López-Martínez R, Méndez-Tovar LJ, Bonifaz A, Arenas R, Mayorga J, et al (2013) Update on the epidemiology of mycetoma in Mexico. A review of 3933 cases. Gac Med Mex 149 (5) 586–92. 24108347

[13] Develoux M, Dieng MT, Kane A, Ndiaye B (2003) Management of mycetoma in West-Africa. Bull Soc Pathol Exot 96: 376–382. 15015843

[14] Lopez Martinez R, Mendez Tovar LJ, Lavalle P, Welsh O, Saul A, et al (1992) Epidemiology of mycetoma in Mexico: study of 2105 cases. Gac Med Mex 128: 477–481. 1308000

[15] Fahal AH, Sabaa AH (2010) Mycetoma in children in Sudan. Trans R Soc Trop Med Hyg 104: 117–121. 19716573 10.1016/j.trstmh.2009.07.016

[16] Ahmed AO, Mukhtar MM, Kools-Sijmons M, Fahal AH, de Hoog S, et al (1999) Development of a species-specific PCR-restriction fragment length polymorphism analysis procedure for identification of Madurella mycetomatis. J Clin Microbiol 37 (10) 3175–8. 10488173 10.1128/jcm.37.10.3175-3178.1999 PMC85521

[17] EL Shamy ME, Fahal AH, Shakir MY, Homedia MMA (2012) New MRI Grading System for the Diagnosis and Management of Mycetoma. Trans R Soc Trop Med Hyg 106 (12) 738–42. 22981317 10.1016/j.trstmh.2012.08.009

[18] Fahal AH, El Sheik H, Homeida MMA, Arabi E, Mahgoub ES (1997) Ultrasonographic imaging of mycetoma. Br J Surg 84 (8) 1120–1122. 9278658

[19] Abd El Bagi ME (2003) New radiographic classification of bone involvement in pedal mycetoma. Am J Roentgenol 180 (3) 665–668. 12591671 10.2214/ajr.180.3.1800665

[20] EL Hassan AM, Fahal AH, EL Hag IA, Khalil EAG (1994) The pathology of mycetoma: Light microscopic and ultrastructural features. Sudan Med J 32: 23–45.

[21] Zein HA, Fahal AH, Mahgoub el S, El Hassan TA, Abdel-Rahman ME (2012) Predictors of cure, amputation and follow-up dropout among patients with mycetoma seen at the Mycetoma Research Centre, University of Khartoum, Sudan. Trans R Soc Trop Med Hyg 106: 639–644. 22854685 10.1016/j.trstmh.2012.07.003

[22] Ahmed AOA, Adelmann D, Fahal AH, Verbrugh HA, Van Belkum A, et al (2002) Environmental occurrence of Madurella mycetomatis, major agent of human eumycetoma in Sudan. J Clin Microbiol 40 (3) 1031–1036. 11880433 10.1128/JCM.40.3.1031-1036.2002 PMC120253

[23] van Hellemond JJ, Vonk AG, de Vogel C, Koelewijn R, Vaessen N, et al (2013) Association of eumycetoma and schistosomiasis. PLoS Negl Trop Dis 23;7 (5) e2241 doi: 10.1371/journal.pntd.0002241 10.1371/journal.pntd.0002241 PMC3662663 23717704

[24] el Hassan LA, Khalil EA, el-Hassan AM (2002) Socio-cultural aspects of leprosy among the Masalit and Hawsa tribes in the Sudan. Lepr Rev 73: 20–28. 11969123

[25] Myint T, Thet AT, Htoon MT, Win M (1992) A comparative KAP study of leprosy patients and members of the community in Hlaing and Laung-Lon townships. Indian J Lepr 64: 313–324. 1431320

[26] Crook N, Ramasubban R, Samy A, Singh B (1991) An educational approach to leprosy control: an evaluation of knowledge, attitudes and practice in two poor localities in Bombay, India. Lepr Rev 62: 395–401. 1784155 10.5935/0305-7518.19910046

[27] Fahal AH, Elkhawad AO (2013) Managing mycetoma: guidelines for best practice. Expert Rev Dermatol 8 (3) 301–307.

[28] Fahal AH (2010) Management of mycetoma. Expert Rev Dermatol 5 (1) 87–93.

